# High CD49f expression is associated with osteosarcoma tumor progression: a study using patient-derived primary cell cultures

**DOI:** 10.1002/cam4.249

**Published:** 2014-05-07

**Authors:** Patrice Penfornis, David Z Cai, Michael R Harris, Ryan Walker, David Licini, Joseph D A Fernandes, Griffin Orr, Tejaswi Koganti, Chindo Hicks, Spandana Induru, Mark S Meyer, Rama Khokha, Jennifer Barr, Radhika R Pochampally

**Affiliations:** 1 Cancer Institute, University of Mississippi Medical CenterJackson, Mississippi; 2 Center for Stem Cell Research and Regenerative Medicine, Tulane UniversityNew Orleans, Louisiana; 3 Department of Orthopedics, Oschner Medical CenterNew Orleans, Louisiana; 4 Ontario Cancer InstituteToronto, canada; 5 Department of Orthopedic Surgery, University of Mississippi Medical CenterJackson, Mississippi; 6 Department of Biochemistry, University of Mississippi Medical CenterJackson, Mississippi

**Keywords:** Cancer stem cell, CD49f, integrin, osteosarcoma, tumor-initiating cell, tumor progression

## Abstract

Overall prognosis for osteosarcoma (OS) is poor despite aggressive treatment options. Limited access to primary tumors, technical challenges in processing OS tissues, and the lack of well-characterized primary cell cultures has hindered our ability to fully understand the properties of OS tumor initiation and progression. In this study, we have isolated and characterized cell cultures derived from four central high-grade human OS samples. Furthermore, we used the cell cultures to study the role of CD49f in OS progression. Recent studies have implicated CD49f in stemness and multipotency of both cancer stem cells and mesenchymal stem cells. Therefore, we investigated the role of CD49f in osteosarcomagenesis. First, single cell suspensions of tumor biopsies were subcultured and characterized for cell surface marker expression. Next, we characterized the growth and differentiation properties, sensitivity to chemotherapy drugs, and anchorage-independent growth. Xenograft assays showed that cell populations expressing CD49f^hi^/CD90^lo^ cell phenotype produced an aggressive tumor. Multiple lines of evidence demonstrated that inhibiting CD49f decreased the tumor-forming ability. Furthermore, the CD49f^hi^/CD90^lo^ cell population is generating more aggressive OS tumor growth and indicating this cell surface marker could be a potential candidate for the isolation of an aggressive cell type in OSs.

## Introduction

Osteosarcoma (OS) is the most common form of malignant bone cancer in adolescents and young adults. Approximately 400 cases are reported in the United States yearly and remains the second leading cause of cancer-related deaths in children and young adults 1,2 with 20% diagnosed as metastatic among patients less than 20 years of age 2. The treatments most commonly used on OS patients have not changed much over the last 30 years and have minimal effect on patients with the metastatic form of the disease 3–6.

In vitro cell cultures derived from human primary tumors are commonly used for multidrug resistance screening 7, proteomics, and the investigation of mechanisms underlying regulatory pathways 8. There are currently a limited number of established human OS cell cultures that have been in use for over three decades. The prolonged culture conditions may have lost many of the qualities that made them satisfactory in vitro models; due to replicative senescence or mutations associated with continued culture propagation 9,10. Compounding the problem, many of these cultures have been immortalized in vitro, adding significant mutations to cell cultures that may already be too far separated from the original tumors. In light of these issues, it is clear that newer in vitro models would be a valuable resource for those who are seeking novel therapeutic options for bone cancers. In this study, we propose to use cell lines derived from patients to develop model for OS tumor initiation and progression.

CD49f is encoded by the integrin α6 gene (ITGA6). CD49f associates with integrin β1 chain to form VLA-6 and with integrin β4 chain to form TSP180, both known to function as laminin receptors 11. Mouse knock out model and human genetic mutation studies demonstrate that CD49f plays an important role in cell adhesion 12,13. In addition, recent studies have pointed out the role of CD49f in the retaining of stemness in mesenchymal stem cells (MSCs) as well as tumor initiation and metastasis of various cancers 14–17.

Studies to identify the OS cancer stem cells (CSCs) or tumor-initiating cells (TICs) highlight a number of available techniques used to isolate and enrich TICs: (1) the sphere culture assay (or sarcosphere assay) 18; (2) cell sorting for CD133, high-ALDH activity, SP cells, or CD117 in combination with Stro-1 19–22; (3) identification of cells that express the embryonic stem cell gene Oct-4 23; and (4) resistance to chemotherapeutic drugs using U2OS and MG63 cell lines 24,25. The above studies shed light on the tumor initiation of OS, however, not much is known about the OS progression. The purpose of this study is to (1) derive several new human cell cultures from primary OS tumors, (2) characterize their morphological and phenotypic traits-like cell surface markers profile and growth characteristics, (3) isolate and evaluate a subpopulation of cells with tumor-initiating and/or progression properties from primary human OS cell lines. We demonstrate that OS cells expressing CD49^hi^/CD90^lo^ exhibit aggressive tumor progression phenotype and thus CD49f can be used as a candidate marker.

## Material and Methods

### Cell culture

The KHOS cell line was obtained from the American Type Culture Collection (ATCC, Manassas, VA) and incubated at 37°C, 5% CO_2_ in culture with Dulbecco's minimum essential medium (DMEM) (Invitrogen, Carlsbad, CA), supplemented with 10% fetal bovine serum (FBS) (Atlanta Biologicals, Lawrenceville, GA), 100 units/mL penicillin, and 100 *μ*g/mL streptomycin (Invitrogen). When adherent cultures reached 80% confluency, cells were lifted with 0.25% Trypsin-EDTA (Invitrogen), and either passaged or frozen with media supplemented with 10% DMSO (Fisher Scientific, Pittsburgh, PA).

Four other OS cell cultures, BCOS, RFOS, RLOS, were patient derived from biopsies obtained from Ochsner Medical Center, New Orleans, LA in accordance with the protocol approved by the Tulane University Health Sciences Center and Ochsner Medical Center Institutional Review Board, and established in vitro following the previously mentioned protocol 26. KRSOS is patient-derived biopsy from UMMC, Jackson-MS, in accordance with the protocol approved by the UMMC's Institutional Review Board, and established in vitro. Portions of patient tumors were mechanically dissociated with a scalpel into 1 mm pieces, disassociated with collagenase and plated in 20 mL of DMEM (Invitrogen), 10% FBS (Atlanta Biologicals), 1% Penicillin-Streptomycin (Invitrogen). The plates were maintained at 37°C in humidified 5% CO_2_ and 95% air, and the media was changed every 2–3 days. Once cells reached 70–80% confluency they were lifted using 0.25% Trypsin-EDTA (Invitrogen) and frozen stock vials for each of the cell cultures were stored in liquid nitrogen. As a control cell line, KHOS was used, a human OS cell line obtained from ATCC. This cell line was derived from HOS (ATCC CRL-1543) by transformation using Kirsten murine sarcoma virus (Ki-MSU). They form tumors in nude mice, have high plating efficiency in soft agar and also display high saturation density 27.

#### Proliferation assays

Cells were seeded in 24-well plates at a density of 10^4^ cells per well at passage 3. Numbers of live cells were counted every 24 h for 6 days using Trypan blue dye exclusion and a hemocytometer.

#### Drug sensitivity studies

Drug sensitivity assays were done in triplicate on 24-well plates seeded at passage 3 and a density of 10^4^ cells/cm^2^. Drugs used were doxorubicin (Sigma, Saint Louis, MO) at concentrations of 0.1, 1, and 10 *μ*g/mL, cisplatin (Sigma) at 1, 10, and 100 *μ*g/mL, and a combination of the two drugs at half of the previously described concentrations (0.05, 0.5 *μ*g/mL; 0.5, 5 *μ*g/mL; and 5, 50 *μ*g/mL, respectively). Cells were treated with the drug-containing media and incubated for 48 h. A proliferation assay using Vybrant (3-(4,5-dimethylthiazol-2-yl)-2,5-diphenyltetrazolium bromide) MTT (Invitrogen) was then performed according to the manufacturer's protocol. Briefly, MTT was dissolved in DMEM culture media and 125 *μ*L was added to each well and incubated for 2 h. Media was then removed from the wells and 300 *μ*L of DMSO was added to dissolve the substrate. Absorbance readings were taken at 570 nm on a FluoSTAR Optima spectrophotometer.

### Differentiation

Cells were plated in 12-well plates at a density of 5 × 10^4^ cells per well. Once 100% confluency was reached, three wells were treated with control media (DMEM 10% FBS), three wells were treated with osteogenic differentiation media (1 nmol/L dexamethasone, 20 mmol/L β-glycerolphosphate, and 50 *μ*mol/L l-ascorbic acid 2-phosphate; Sigma-Aldrich), and three wells were treated with adipogenic differentiation media (0.5 *μ*mol/L dexamethasone, 0.5 *μ*mol/L isobutylmethylxanthine, 50 *μ*mol/L indomethacin; Sigma-Aldrich) 28. Cells were maintained for 21 days, and were then fixed in 10% buffered formalin solution (Sigma-Aldrich) and stained with Alizarin Red (Sigma-Aldrich) for osteogenesis and oil red O stain (Sigma-Aldrich) for adipogenesis 28.

### Subcutaneous injections to monitor tumor growth and development

Between 1 × 10^5^ and 3 × 10^6^ cells were injected in the in vivo experiments depending on experimental limitations and sorting procedures. Prior to injection, cells were trypsinized and collected from adherent cultures. Cells were washed three times in PBS and resuspended in 200 *μ*L of Hanks-buffered saline solution (HBSS; Invitrogen). Cells were maintained on ice before injection to female nu/nu mice. Tumor growth was monitored at regular intervals using digital calipers and average tumor volume was calculated using the following formula: ^4^/_3_ π a b^2^.

Tumor samples were fixed in 10% formalin and stained with hematoxylin–eosin for further histological characterization. All in vivo experiments were carried out in accordance with and approval of Tulane's and University of Mississippi Medical Center's Institutional Animal Care and Use Committee.

### FACS analysis and CD49f enrichment

For 30 min at room temperature, 5 × 10^6^ cells derived from tumors were dissociated with trypsin, resuspended in PBS, and incubated with a CD49f-conjugated antibody to PE-Cy5 (BD Biosciences, San Jose, CA, clone GoH3). Cell suspensions were then washed three times in PBS and percentage of positively expressed cells was measured using a Cytonomics FC500 flow cytometer (Beckman Coulter, Miami, FL). Cells that were positive for CD49f were plated back to promote adherence. After 24 h, nonadherent cells were washed away with PBS and fresh media was added. All cell cultures were phenotyped at passage 2 or later using the Beckman Coulter Epics FC 500 Flow Cytometer with CXP Software (Beckman Coulter). Antibodies were obtained from Beckman Coulter (Atlanta, GA) MBL International (Woburn, MA), BD Biosciences (San Jose, CA), and EBiosciences (San Diego, CA). Cell surface markers tested for are shown in Table2, the phenotyping was performed using the protocol and description of antibodies as described previously by our laboratory 29.

### Amiloride treatment

KHOS cells were treated overnight with 0–250 *μ*mol/L of amiloride (Sigma-Aldrich) following the protocol described by Demetriou et al. 30. After 24 h, plates were washed three times with PBS and adherent cells were subsequently trypsinized and counted before being prepared for in vivo injections.

### Lentiviral transfection

For lentiviral transfection of both KHOS-GFP and KHOS-CD49f shRNA GFP, KHOS cells were plated at densities in triplicate of 3 × 10^3^, 2 × 10^3^, 1 × 10^3^, and 5 × 10^2^ in 12-well plates in 10% FBS DMEM media. Once adhered, media was refreshed and supplemented with 7 *μ*g/mL polybrene (Millipore, Billerica, MA). GFP control lentivirus, vector pNL-EGFP/CMV-WPREdU3, was kindly obtained from the Louisiana State University Vector Core Facility (http://www.medschool.lsuhsc.edu/genetherapy/vector_intro.aspx), who also packaged the obtained CD49f shRNA pGIPZ GFP containing lentiviral plasmid (ID:NM_000210, Open Biosystems, Huntsville, AL) for lentiviral knockdown expression.

Lentivirus was added at multiplicity of infection (MOI) between 7 and 90 to optimize the transfection using GFP expression as a guide. Cells were incubated overnight and the viruses were removed following the replacement of transfection media with standard media. Cells were incubated for 72 h before being lifted by trypsinization and sorted using FACS for positive GFP cells.

### Western blot

Cell pellets were prepared by centrifugation at 500*g* for 4 min and lysed in RIPA buffer (Santa Cruz Biotechnologies, Santa Cruz, CA) containing protease inhibitor cocktail for 1 h on ice. A protein concentration was then determined using a BCA Protein Assay Kit (Pierce Biotechnology, Rockford, IL). For denatured reduced protein analysis, 100 μg of protein lysate was prepared. Samples were heated for 10 min at 70°C with LDS Buffer (Invitrogen), fractionated by a NuPage 4–12% Bis-Tris sodium dodecyl sulfate-polyacrylamide gel electrophoresis (Invitrogen). The gel proteins were then transferred to a Millipore Immobilon-P PVDF membrane (GenHunter Corporation, Nashville, TN) by electroblotting and the membrane was blocked overnight at 4°C in PBS containing 0.05% Tween 20 (Sigma-Aldrich) and 5% nonfat dry milk (Santa Cruz).

Membranes were then incubated with primary mouse monoclonal antibody diluted 1:1000 for CD49f (clone 7H164, US Biologicals, Marblehead, MA) in TBS plus 1% nonfat milk overnight with agitation. After three washes, the secondary antibody goat anti-mouse HRP (Chemicon, Temecula, CA) diluted 1:10,000 was then added in similar conditions, and incubated for 1 h at room temperature. Three washes of TBS were performed before exposure using an ECL Western Blotting Substrate (Pierce, Rockford, IL).

### Migration assay

In serum-free media, 1 × 10^5^ cells/mL single cell suspensions of both KHOS-GFP and KHOS-GFP-shCD49f were prepared; 5 × 10^4^ cells were loaded into the upper well of a BD Falcon HTS FluoroBlok 24-Mulitwell Insert System (BD Biosciences) with 8 *μ*m pores. DMEM containing 10% FBS was added in the lower wells serving as a chemoattractant. Cells were then incubated overnight and GFP fluorescence was measured at 485 nm excitation and 520 nm emission in an OPTIMA FLUOstar plate-reader (BMG Labtech, Cary, NC). To further quantify, three randomly selected fields were chosen per well and the fluorescent migrated cells were counted.

### Nonadherent clonogenicity assay (sarcosphere assay)

Single cell suspensions were collected and 2 × 10^3^ cells were plated in each well of a Nunc Low-Cell Binding (Nunc, Rochester, NY) six-well plate in normal media. Cells were incubated for 12 days before being transferred to adherent plates to allow for adherence for 24 h. Colonies were then stained with Crystal Violet solution (Sigma-Aldrich) and colonies containing more than 200 cells were quantified. Clonal density was used as described by Patrawala et al. 31 and nonadherent plates were used as substitutes for agar plating.

### Gene expression assays

Total RNA was isolated from the second passage of cultured cells using Rneasy kit according to manufacturer's protocol (Qiagen, Valencia, CA). To synthesize double-stranded cDNA, 8 *μ*g of total RNA was used (Superscript Choice System; Invitrogen). Following cDNA synthesis, the sample was purified by phenol/chloroform extraction and concentrated by ethanol precipitation. In vitro transcription was used to produce biotin-labeled cRNA (BioArray HighYield RNA Transcription Labelling Kit; Enzo Diagnostics, Farmingdale, NY). The biotinylated cRNA from KHOS, RFOS, RLOS, and BCOS was cleaned (RNAeasy Mini Kit; Qiagen), fragmented, and hybridized on the Affymetrix microarray chips (HUG133 plus 2.0 gene chip Affymetrix, Santa Clara, CA). The biotinylated cRNA from KRSOS was fragmented and hybridized on Agilant Platform microarray (Surechip G3v2). The individual samples were normalized as per manufacturer's recommendation and as described earlier 32,33. Four cell lines (BCOS, KHOS, RLOS, RFOS) expression profiling was performed on Affymetrix platform HGU133 plus 2.0 gene chip. For unsupervised analysis of sample clustering for the “stemness,” expression signatures were obtained from Song et al. 34. Probes for stemness genes and their average expression values from two samples for each cell line were obtained from microarray analysis for BCOS, KHOS, RLOS, and RFOS groups. After further processing and removing duplicates, we ended up with 116 genes. Expression values for the set of novel 116 genes were obtained for KRSOS cell line from Agilent platform. We performed unsupervised analysis based on hierarchical clustering using the complete linkage method and the Pearson correlation coefficient as the measure of distance between pairs of genes. Gene expression data for all the groups were normalized using median normalization method. Hierarchical clustering was performed to generate a heatmap showing the expression pattern for 116 genes among all the cell lines. Clustering module from Gene Pattern platform was used for this analysis 35.

## Results

### Gross anatomy, histological features, and imaging studies of primary tumors

Four OS cell cultures were generated; the demographics of cell cultures are presented in Table1. First, we report the gross anatomies and histological features of the primary tumors.

**Table 1:** Demographics of patients from which the osteosarcoma cell cultures are derived.

Cell line	Gender	Race	Age	Location	Histological characterization
BCOS	Male	Caucasian	25	Radius	High-grade osteosarcoma
RFOS	Female	Caucasian	18	Humerus	High-grade osteosarcoma with areas of necrosis
RLOS	Female	Caucasian	38	Tibia	High-grade osteosarcoma with chondroid differentiation
KRSOS	Female	African American	12	Distal femur	High-grade osteosarcoma

### BCOS

**Figure 1:**
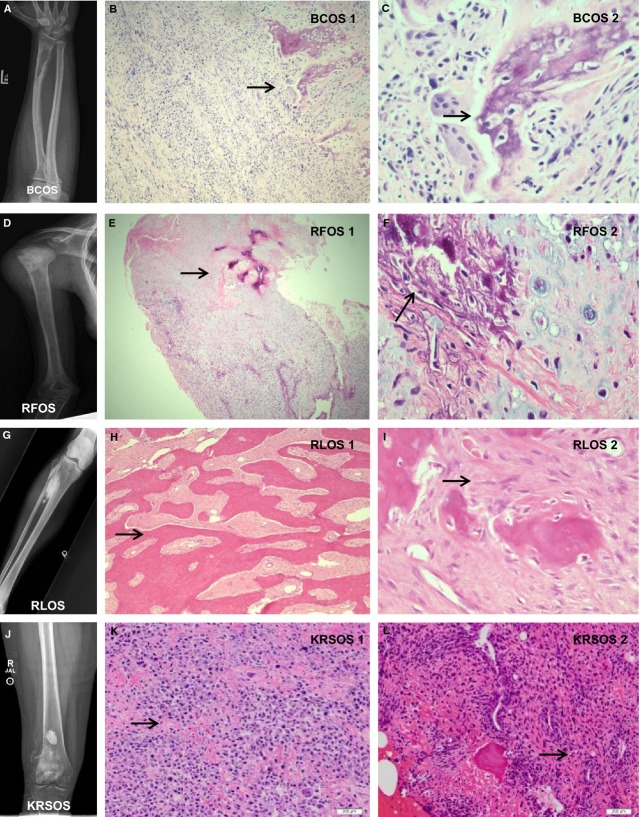
Gross anatomy, histological features, and imaging studies. (A, D, G, and J) The X-ray photographic images of the tumor localization of BCOS, RFOS, RLOS, and KRSOS, respectively. (B, E, H, K, and L) The low-magnification H&E staining images of BCOS, RFOS, RLOS, and KRSOS, respectively. (C, F, and I) The high-magnification H&E staining images of BCOS, RFOS, and RLOS, respectively.

This tumor was derived from a 25-year-old male from the distal radius. The tumor grossly had rubbery, soft, light tan to pink tissue in total size of 2.3 × 1.7 cm. The radiographic images shown in Figure1A (BCOS) shows an irregular oval lytic lesion within the distal radius with adjacent periosteal reaction and elevation consistent with a Codman's triangle. The vast majority of the lesion was a high-grade spindle cell sarcoma with nuclear atypia and hypercellularity with common mitoses. This is demonstrated in Figure1B (BCOS 1). Areas between the spindle cells were filled with pink material consistent with osteoid. In the high-power image (Fig.1C, BCOS 2) bone formation with numerous osteoblasts rimming this new bone is shown. The osteoblasts also had features of atypia, making this a high-grade spindle cell sarcoma with features pointing toward OS.

## RFOS

This tumor was from the proximal metaphyseal region of a right humerus in an 18-year-old female. The tumor was a 15 × 6.5 cm gray to light yellow mass with a few soft rubbery tan to pink areas. The tumor invaded the humeral periosteum with periosteal reactive changes (Fig.1D RFOS). The lesion was a high-grade cell tumor with areas of necrosis. Figure1E (RFOS 1) shows the tumor bone in the top central area of the slide. Figure1F (RFOS 2) is high-power microscopy demonstrating the chondroid tissue adjacent to the tumor bone. Also there were significant areas of chondroid tissue intermixed within the lesion. These findings were consistent with a high-grade OS.

## RLOS

This tumor was derived from a moderately well defined 8 × 3.2 cm firm mass involving the proximal tibia of a 38-year-old female. The cut surface of the tumor was light yellow to gray. The tumor projected about 1.5 cm from the cortical bone into the periphery, but blended into the cortex along the cortical margin (Fig.1G, RLOS). There were well-formed parallel bony trabeculae (Fig.1H, RLOS 1) surrounded by a cellular spindle cell proliferation with minimal atypia, consistent with parosteal OS. High-power microscopy is demonstrated in Figure1I (RLOS 2).

## KRSOS

This tumor was derived from a 6.5 × 5.2 cm tumor in the distal femur of a 12-year-old female. Its gross section revealed a tan-red, variegated, ill-defined intramedullary tumor centered on the femoral metaphysis appearing to invade through the lateral and posterior cortex into the overlying soft tissue (Fig.1J). Histologic sections show a proliferation of malignant spindle to epithelioid cells with marked nuclear pleomorphism, clumped chromatin, large nucleoli, and moderate amphophilic cytoplasm Figure1K–L. Abundant lace-like osteoid and woven bone surrounds individual and groups of cells consistent with high-grade conventional osteoblastic OS.

## Osteosarcoma cell cultures express mesenchymal markers

Cells obtained were analyzed by flow cytometry to assess specific cell surface markers (Table2). The high expression of CD44, CD90, and CD105 as well as the low expression of CD117 and CD49f confirms the mesenchymal origin of the cell cultures 36. The former markers commonly found in MSCs and the latter are indicative of hematopoietic stem cells 29,36. This confirms the cell cultures generated are mesenchymal, the origins of OSs.

**Table 2:** Flow cytometric assays to test cell surface markers expression for mesenchymal phenotype.

	RFOS	RLOS	BCOS	BCOS TD	KRSOS	KRSOS TD	KHOS
CD 44	5	8	99	85	100	11	98
CD 90	9	12	99	13	100	7	4
CD 49b	1	4	13	96	92	8	90
CD 105	17	22	100	98	99	5	97
CD 117	1	2	1	5	1	8	1
CD 49f	10	17	39	91	11	64	87

Relative expression in OS cell lines at early passage and posttransplantation in nu/nu mice (TD).

## Growth and differentiation characteristics of the derived cell cultures

**Figure 2:**
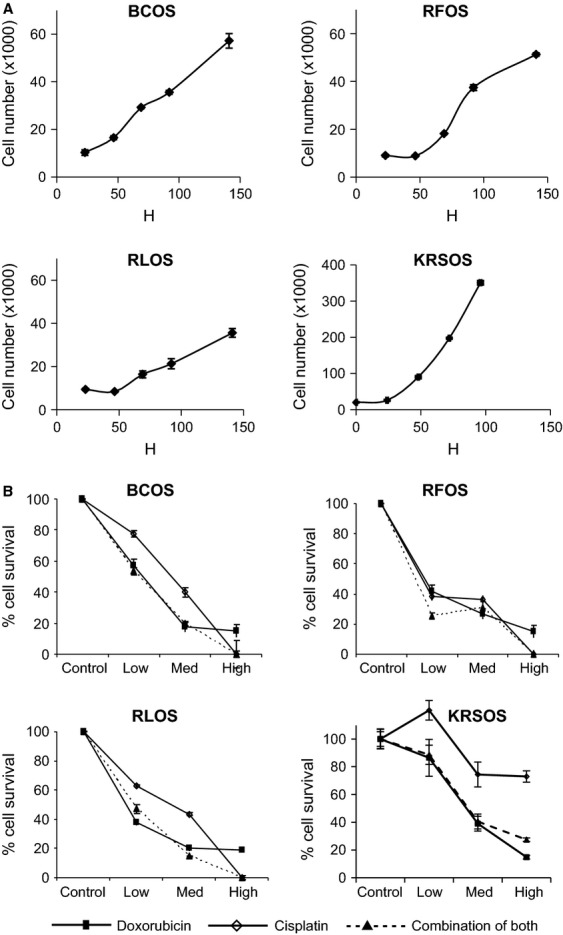
Growth and drug sensitivity assays of OS cell cultures. (A) Growth properties of OS cell cultures, BCOS, RFOS, RLOS, and KRSOS, respectively. (B) Relative drug sensitivity to doxorubicin (plain square), cisplatin (open diamond), or combination of both (plain triangle) of BCOS, RFOS, RLOS, and KRSOS, respectively.

First, we performed the proliferation assays of subcultured cell cultures at passage 3. All the cell cultures were plated in a 24-well plate at similar density and proliferation was determined by cell count using hemocytometer as described in 37 over 6 days. The growth curves indicate that the cell lines have varying growth patterns (Fig.2A). KRSOS was fastest growing cell line over 6 days with a doubling time of 22 h. BCOS and RFOS and have intermediate doubling times of 61 and 34 h, respectively, and RLOS was our slowest growing cell culture with doubling times of 98 h. The phase-contrast pictures of actively replicating cells are represented in Figure S1.

One of the diagnostic properties of OS is differentiation into osteoblasts/deposit mineral. At passage 3, these cells cultures were assessed for their ability to differentiate into mineralized cells by treatment with osteogenic media containing factors that trigger mineral deposition leading to preosteoblastic phenotype in potential osteocyte precursors, a behavior indicative of OS cells and MSCs. A separate assay was done using adipogenic media containing factors that trigger adipose cell differentiation; behavior demonstrated by bone marrow-derived MSCs, but not OS cells. As shown in Figure S2, we observed high osteogenic differentiation of all cell cultures and the lack of differentiation into adipocytes. Only a few cells were positive for oil red O. The data indicate that there may be a very small number of stray MSC's and this can be expected, as these cells are at a very low passage, taken from bone sections of OS patients and the slow-growing MSCs will be outgrown by the fast growth of malignant cells.

## Drug sensitivity assays

Next, we evaluated the sensitivity of cell cultures for the two common chemotherapeutic drugs: doxorubicin and cisplatin. Cell cultures were plated in 24-well plates and treated with three incremental concentrations of both drugs (Fig.2B). Interestingly, the KRSOS line appears to be more resistant to doxorubicin and cisplatin. RFOS appears to be the most sensitive of OS cell cultures. The chemoresistance of OS tumors are not well understood therefore, these cell cultures would provide excellent tools for studying resistance. Table3 summarizes the cell culture properties of all three cell cultures.

**Table 3:** Summary of cell culture characteristics of OS cell lines.

Cell line	Primary diagnostic	Doubling time at passage 3	Accumulated population doubling	Relative drug sensitivity	Sarcosphere formation	Total passage tested
BCOS	Osteosarcoma	61 h	>100	Moderate	Yes	>20
RFOS	Osteosarcoma	34 h	>45	High	ND	>11
RLOS	Parosteal osteosarcoma	98 h	>50	Moderate	ND	>12
KRSOS	Osteosarcoma	22 h	>100	Low	Yes	>36

## Primary osteosarcoma tumors with higher CD49f^hi^/CD90^lo^ expression have greater tumor implantation and progression

Although several studies have reported characterizing OS initiation and progression, a major shortcoming of such studies has been the use of cell lines that have been cultured for several generations. In this study, we used primary human OS cells in an immunodeficient mouse model. First, the primary OS cells were obtained from primary tumors and digested and processed in three ways: (1) expansion and maintenance in vitro through previously described cell culture techniques, (2) initial cell-surface marker characterization, and (3) subcutaneous injection into mice for transplantation assays. A preliminary subcutaneous xenograft using established cell lines indicated that CD49f expression levels are associated with tumor formation. As shown in Table4, the cell lines with a CD49f^hi^/CD90^low^ phenotype are associated with tumor formation. With these data, we tested the hypothesis that the cell population with CD49f^hi^/CD90^lo^ phenotype carries the tumor progression property of human OS.

**Table 4:** Cell surface phenotypes in various established cell lines.

Cell line	KHOS	SJSA-1	MG-63	U20S	BCOS	RFOS	RLOS	KRSOS
CD49f	+	+	+	+	−	−	−	+
CD90	−	−	+	+	+	+	+	+
Subcutaneous tumor implantation	+	+	−	−	−	−	−	+

Initial observations with subcutaneous tumor implantation rates of established cell lines showed a correlation of CD49f^hi^ and CD90^lo^ cell lines having a greater tumor implantation and occurrence rate. Threshold of 50% measured expression by FACS analysis, as above 50% to be (+) and below 50% to be (−).

First, the role of CD49f expression in tumor implantation and survival was studied by using a cell lines derived from a primary OS. We tested if the CD49f expression levels increased post implantation. We used BCOS and KRSOS cell lines for phenotype characterization posttransplantation. Posttransplantation, the tumors were harvested and phenotyped as described in the Material and Methods section. The posttransplantation cell population has shown significant increase in the CD49f^hi^ population, suggesting a role of CD49f-positive cells in tumor survival (Table2).

## CD49f is critical for OS tumor proliferation

**Figure 3:**
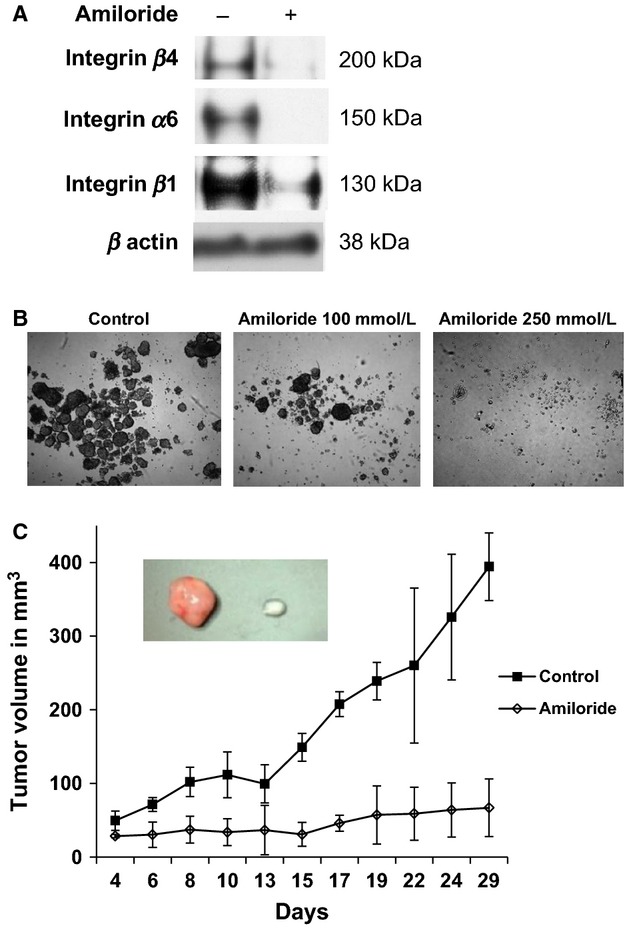
Amiloride blocks CD49f expression and reduces tumor growth. (A) Western blot assays to determine CD49f (integrin α6) expression in KHOS cell lines in the presence or absence of amiloride. (B) Sarcosphere formation by KHOS cell lines in the presence or absence of amiloride. (C) KHOS tumor growth in vivo in presence of amiloride. Growth curves of 2 × 10^6^ KHOS cells treated with amiloride and then transplanted into nu/nu mice. Insert illustrates ex vivo tumor size obtained after 29 days after injections of KHOS control cells (left) and amiloride-pretreated KHOS cells (right).

**Figure 4:**
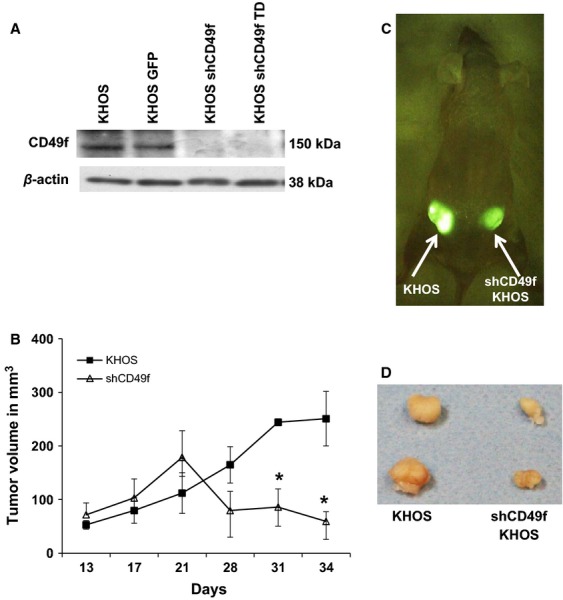
shCD49f inhibition of CD49f reduces tumor growth. (A) Western blot assays for CD49f in KHOS and shCD49f KHOS cells. (B) 1 × 10^6^ KHOS and 3 × 10^6^ shCD49f cells were injected subcutaneously into nu/nu mice, respectively, and tumor measurements were taken up to day 34 (*P < 0.05). (C) In vivo visualization of KHOS GFP on the left side and KHOS GFP shCD49f on the right side. (D) Representative ex vivo tumor pictures at day 34 after injections. Harvested tumors illustrate size difference between groups.

The next step is to better understand the role of CD49f in tumor progression of OS. To test this, two approaches were taken. First approach was to use amiloride, a common potassium-sparing diuretic, which has been shown to regulate CD49f expression levels 30. We chose the KHOS cell line for this study due to its high-CD49f and low-CD90 cell-surface marker expression phenotype. A western blot assays confirmed that 24 h post-amiloride treatment (250 *μ*g/mL) showed a decrease in CD49f expression (Fig.3A). When tested the sarcosphere formation of KHOS cells treated with amilioride, as shown in Figure3B, the number of sarcospheres decreased significantly with increasing concentration of amiloride. Next, we subcutaneously injected 2 × 10^6^ amiloride-treated and untreated KHOS cells into nu/nu mice. Tumors were observed within the first week for the untreated KHOS group, whereas the amiloride-treated group showed minimal tumor initiation and progression (Fig.3C). KHOS control tumors continued to grow for 3 weeks and then followed a regression pattern similar to CD49f^lo^ cell lines. We observed that tumor growth was completely inhibited in the amiloride-treated group (Fig.3C). Next, we sought to directly inhibit CD49f expression using shRNA against CD49f as shown by Cariati et al. 38. The KHOS cell line was selected for these experiments because over 90% of KHOS cells expressed the CD49f^hi^/CD90^lo^ phenotype. Lentiviral shRNA to CD49f was transduced with GFP expression. Wild-type KHOS cells transduced with a GFP vector were chosen to serve as the control 39. Following transfection, FACS analysis for CD49f expression showed a threefold decrease in the KHOS-GFP shCD49f group when compared to its respective control KHOS-GFP and KHOS (naked) cell lines (data not shown). Under general cell-culture conditions, growth kinetics of all three cell lines appeared similar. The KHOS-GFP and KHOS-GFP-shCD49f groups were observed to have an enriched and diminished CD49f expression, respectively, and were selected for additional in vivo studies (Fig.4). These cells were expanded in vitro and 3 × 10^6^ KHOS-shCD49f-GFP and 1 × 10^6^ KHOS-GFP cells were injected subcutaneously into nu/nu mice (*n* = 4). Tumor progression of both cell lines was closely monitored at regular intervals in order to study the effect of CD49f expression and its correlation with tumor implantation and progression in vivo (Fig.4B). Measurements taken after day 30 showed a significant difference (*P* = 0.01) between the two test groups. Both groups showed positive tumor growth until day 30. After day 30, the KHOS-GFP-shCD49f group showed a reduction in tumor size, whereas the KHOS-GFP group was observed to plateau in tumor size, suggesting less aggressive tumor proliferation capability (Fig.4B–D). The tumors clearly showed significant decrease in tumor volume (Fig.4C and D). Interestingly, the intake and take of phase was similar in both CD49f knock down cells and control cells. However, the tumor progression is greatly inhibited, leading to smaller tumors in CD49f knockdown cells (Fig.4B and D). Taken together, the role of CD49f in tumor progression is clearly evident in both sarcosphere and xenograft transplantation assays.

## CD49f inhibition decreases the migratory ability and contact inhibition

Due to the involvement of integrins in the cell's ability to proliferate and interact with its microenvironment, we chose an in vitro migration assay to test the efficiency of CD49f knockdown. KHOS-GFP-shCD49f cells and KHOS-GFP controls were incubated overnight with media containing 10% FBS in the bottom well only. A matrix with 8 *μ*m filter pores served as a barrier for the cells to migrate through, and fluorescence measurements were used to quantify the degree of migration that occurred through the filter.

**Figure 5:**
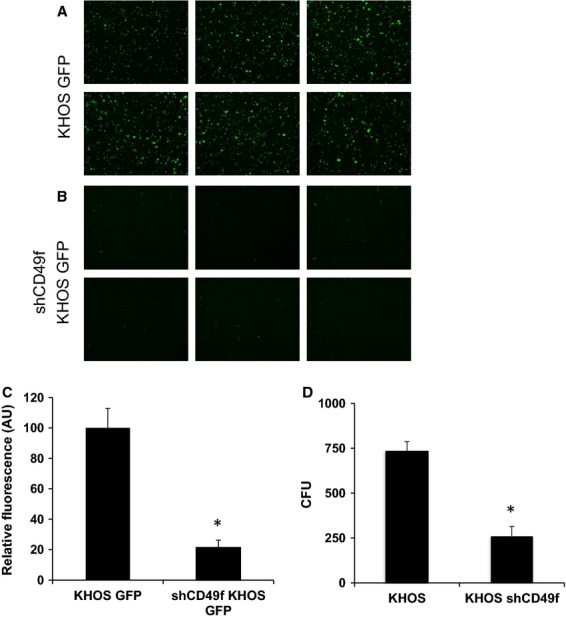
shCD49f inhibition decreases cell migration and sarcosphere formation. (A and B) Fluorescent microscopic images of transwell migration assays of KHOS and KHOS shCD49f cell lines. After 24 h incubation period, fluorescence of cells that have migrated through a 8 μm pore membrane was measured. Pictures representing the migrated cells of either (A) KHOS-GFP or (B) KHOS-GFP-shCD49f cell lines (C). Quantitative analysis of the fluorescence expression of migrating cells. (D) Quantitative numbers of sarcospheres formed in KHOS and KHOS shCD49f cell lines. The bar chart shows the average number of colonies formed originally placed under nonadherent conditions. Colonies were stained with crystal violet and further quantified by counting and a 2.8-fold decreased in colony formation was observed in the KHOS shCD49f group (*P < 0.05).

Figure5A–C illustrates a significant (*P* < 0.002) decrease in migration capability that we observed in the KHOS-GFP-shCD49f cell line. The quantification of migration was further analyzed by counting in a double-blind manner (i.e., by two independent associates). Our data suggest that the KHOS-GFP-shCD49f cell line experienced a significant decrease in its ability to migrate through the barrier, confirming that the CD49f was successfully downregulated through lentiviral transfection (Fig.5C).

Next, we tested the effect of CD49f expression on sarcosphere formation, a commonly accepted characteristic to define TICs/CSCs. KHOS-GFP and KHOS-shCD49f-GFP cells were cultured in nonadherent clonogenicity assay and assayed for sarcosphere formation. Colonies were quantified per group and then averaged. We observed a greater number of well-formed colonies in the KHOS group compared to the KHOS-shCD49f group. Our data suggest a significant (*P* = 0.001) reduction in clonogenicity abilities between the KHOS shCD49f group and the control group (Fig.5D).

## Stemness markers are highly expressed in CD49f^hi^ cells

**Figure 6:**
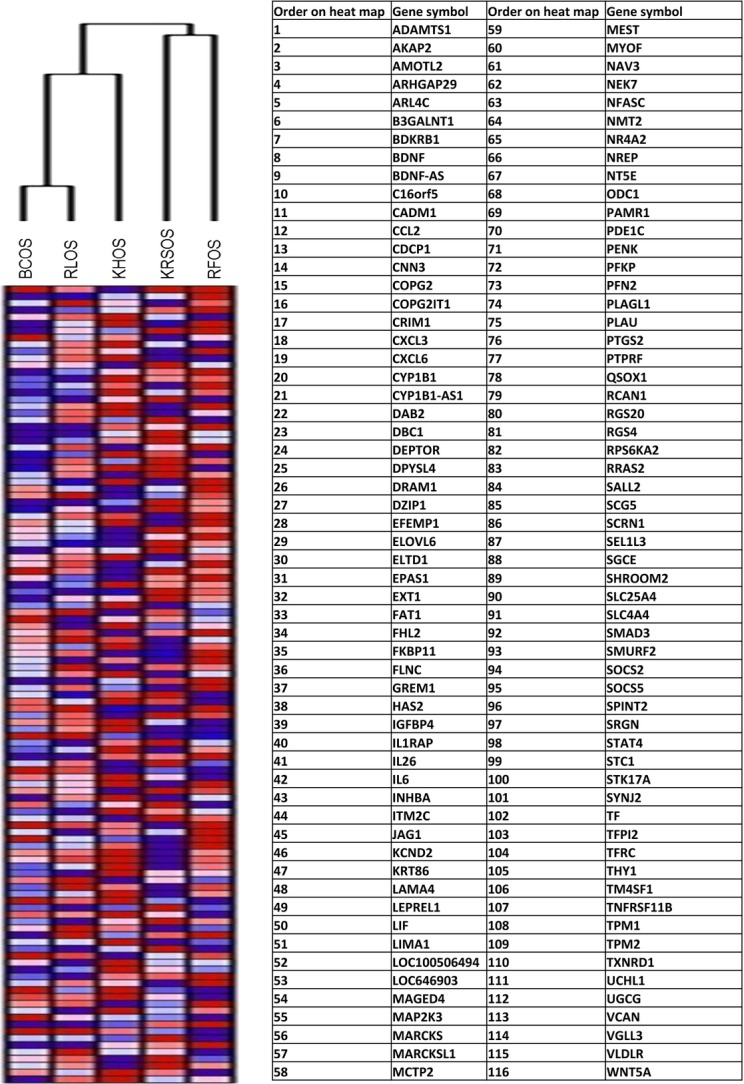
DNA microarray analysis illustrating that cells that express high levels of CD49f (KHOS) also show a greater expression of various stemness/progenitor markers. Four human OS cell lines were analyzed and units are in values of relative fluorescence in relationship with each gene tested. The list of genes is expressed in the table, in the order of their appearance on the heat map.

A DNA microarray analysis was performed on three human OS cell lines. Although our focus was on the CD49f cell surface marker, other potential markers characterizing progenitor cells such as hematopoietic, mesenchymal, and embryonic stem cells were also included in conjunction with CD49f. A hierarchical clustering of the expression data showed that KHOS cell line inherently expressed higher levels of the CD49f markers also expressed a significantly higher level of various progenitor cell markers when compared to two other OS samples. We analyzed the unregulated and downregulated genes according to stemness by hierarchical clustering and pathway analysis using the GenMAPP 2.0 program for three OS cell lines as described by Song et al. 34 (Fig.6). The gene expression data corroborated the in vitro characterization of the cell lines. As expected, KRSOS clustered closest to KHOS. All cell lines exhibited unique expression profiles with overlap in stemness genes, interestingly on both sides of KHOS, closely resembling their doubling time.

## Discussion

Well-characterized sarcoma cell cultures derived from primary tumors that exhibited both morphological and gene expression profiles closely corresponding to the primary bone tumors are important for several reasons. First, there is no small animal model of spontaneous sarcoma formation that accurately replicates the course of the human disease 40. Transgenic animal models of human disease do not reproduce the histiogenesis of human sarcoma and animal sarcoma differs substantially from human tumors 40. Second, cancer evolves through a process of stepwise accumulation of genetic alterations that result in uncontrolled cell proliferation and a lack of response to normal apoptotic stimuli. Availability of cell cultures that correspond to the events that undergo in OS genesis may serve as important in vitro models 40–42.

In this study, we describe derivation and characterization of four OS cell cultures derived from patients and use of one to understand the role of integrins in OS progression. The histopathological and anatomic characterization of the tumors was performed to confirm the pathology of the tumors. Next, passaged cell cultures were tested for properties like growth rate, cell surface markers, differentiation capacity, and drug resistance. The cell cultures exhibit variable growth and phenotypic properties while sharing the property to differentiate into osteoblasts and expression of mesenchymal markers. In addition, variable drug sensitivity is a major obstacle in developing therapies for OS. The availability of gene expression data for all the cell cultures makes these ideal in vitro models to understand molecular and drug resistance properties. Furthermore, the ability of all the cell cultures to produce mineralized cultures and express variable levels of osteoblast-specific genes is very important.

Furthermore, in continuation of previously published study using BCOS, which showed that CD117 and Stro-1 are associated with OS tumor initiation and metastasis 20,43, we sought out to understand the role of CD49f in tumor progression. Our results demonstrate that there is a positive correlation between a high CD49f expression and aggressive tumor progression. Through phenotyping multiple OS cell lines, it was initially observed that the cells, which expressed the pattern of CD49f^hi^ with CD90^lo^ produced the most aggressive tumors (Table4).

Next, we tested inhibition of CD49f with amiloride, as it has been shown to inhibit a few isoforms of CD49 30. To confirm that amiloride would also decrease expression of CD49f isoform, western blot assays confirmed that CD49f was greatly reduced in the presence of amiloride (Fig.3A). Tumor implantation with amiloride-treated tumor cells suggests that the lower expression of CD49f correlates with a decreased tumorigenicity (Fig.3B). A small percentage of cells underwent apoptosis in response to amiloride treatment but only viable cells were used for the in vivo portion of the study. Amiloride treatment of KHOS cells significantly decreased the tumor progression, however, tumor initiation was comparable between treated and untreated groups (Fig.3C).

To further confirm the role of CD49f in tumor progression, we constructed a lentiviral vector with GFP tagged shRNA against CD49f 38. Upon transfection of this construct into KHOS cell line, the expression studies showed a knockdown CD49f expression levels (Fig.4A) and a concomitant significant reduction in tumor development over a period of 34 days (Fig.4B). Next, to demonstrate the role of CD49f in progression of tumor growth, we injected three times more number of shCD49f cells compare to KHOS control cells. The shCD49f cells have demonstrated decreased proliferative rate and xenograft experiments with equal number of cells as KHOS, did not form any tumors in mice (data not shown). Therefore, in order to further demonstrate the role of CD49f in tumor progression we have injected higher number of cells. The data support the role of CD49f in tumor progression rather than initiation.

Measuring the functional effectiveness using a cell migration assay, our results support a previous study suggesting that CD49f expression is involved in cancer progression 44. The supporting data for CD49f role are; first, colony forming unit assay to determine the proliferative population of the cells lines, which showed more established colonies among the KHOS control group compared with the KHOS shCD49f (Fig.4D) 45. This suggests that the presence of CD49f plays a role in the establishment and progression of cancer cells. In vivo data (Fig.4B) illustrate that limited tumor progression was observed when CD49f was under-expressed or knocked down using shRNA. These data suggest that due to the lack of CD49f antigens being expressed by the cell, the absence of cellular-binding capabilities would further inhibit normal intracellular signals. This demonstrates the importance of cell–cell communication via integrin-mediated intracellular signaling, and that blocking this mechanism might inhibit “normal” tumor progression.

Data from the DNA microarray analysis suggest that the KHOS CD49f^hi^ cell population also expresses a higher level of various other stemness type genes when compared to the two other human OS cell lines that expressed lower CD49f (RFOS and RLOS) (Fig.6). Additionally, the short hairpin lentiviral transfection α6 integrin should be further tested for its efficacy in specifically blocking the expression of the CD49f gene, and not the other CD49 isoforms. Our data supports the notion that the CD49f marker can serve as a promising candidate for isolating a subpopulation that exhibits aggressive sarcomagenesis. Characterizing and successfully isolating this subpopulation of human cells may play an important role in targeting more effective therapies for OS.

Although several OS lines were generated, currently there are less than seven cell lines that are commonly used for OS studies (Table S1). All of the characterization of these new OS cell cultures in this report extends the several reports of primary OS cell line cultures that date back to late 1960s 46, U2OS the cell line generated from primary aggressive OS. Most of the subsequent reports of cell lines derived from OS lines cluster around 1970s 46, U2OS the cell line generated from primary aggressive OS. Most of the subsequent reports of cell cultures derived from OS cultures cluster around 1970s 27,47–50 and most of the earlier studies focused on biochemical and cytogenetic analysis. Adding an additional dimension, this report presents analysis of primary cell cultures with high-resolution microarrays to compare gene expression profiles. The present report expands the available OS cultures derived from adults as opposed currently available cell cultures derived from paediatric cancers. These cell cultures provide a valuable tool to study adult onset bone cancers.

Strengths of the four cell cultures described in this report for in vitro study of sarcoma biology and testing of therapeutic targets relate to (1) origin from adult onset and adolescent onset primary bone cancers, (2) demonstration of cellular propagation through at least 10 passages, (3) documentation of gene expression patterns in the cell cultures at passage 3, and (4) preservation of cell cultures by freezing at passage 3 to provide highly characterized cell cultures for future studies that involve both immortalization, chemo resistance and therapeutic target assays.

## Supporting Information

**Table S1**. Summary of the commercially available OS cell lines. **Figure S1**. Representative phase-contrast microscopic images of the different bone primary cancer-derived cell cultures. (A) BCOS, (B) RFOS, (C) RLOS, and (D) KRSOS. Bar represents 200 *μ*m. **Figure S2**. Differentiation into osteocytes (A, B, C, D) or into adipocytes (E, F, G, H) of the different bone primary cancer-derived cell cultures: BCOS (A, E), RFOS (B, F), RLOS (C, G), and KRSOS (D, H). All differentiation assays have been done at the same passage. Bar represent 200 *μ*m.
