# Supplementary material for: High CD49f expression is associated with osteosarcoma tumor progression: a study using patient-derived primary cell cultures
Source: Cancer Med. 2014 May 7;3(4):796–811. doi: 10.1002/cam4.249 (PMC4303148; doi:10.1002/cam4.249)
Supplement: Supplementary file 1 [file cam40003-0796-sd1.pdf]

**Supplemental figures Legends:**

**Table S1**: Summary of the commercially available OS cell lines.

**Figure S1**:Representative phase contrast microscopic images of the different bone primary cancer derived cell cultures. A) BCOS, B) RFOS, C) RLOS, D) KRSOS. Bar represents 200μm.

**Figure S2**: Differentiation into osteocytes (A,B,C,D) or into adipocytes (E,F,G,H) of the different bone primary cancer derived cell cultures: BCOS (A,E), RFOS (B,F) , RLOS (C,G), KRSOS (D,H). All differentiation assays have been done at the same passage. Bar represent 200μm.

Table S1

| **Cell line** | **Gender, Age, Ethnicity** | **Characteristics** | **References** |
|---|---|---|---|
| **MG-63** | Male, 14 years, Caucasian | Hypotriploid human cell line | Billiau A et al. 1977 (3) |
| **HOS** | Female, 13 years, Caucasian | Mixed, fibroblast and epithelial like cells | McAllister RM et al. 1971 (5) |
| **KHOS** | Female, 13 years, Caucasian | Derivative of HOS transformed with Kirsten sarcoma virus | Rhim JS et al. 1975 (7) |
| **SJSA** | Male, 19 years, African American | Amplification of *mdm2*. and *gli* proto-oncogene. | Roberts WM et al. 1989 (8) Oliner JD et al. 1992 (6) |
| **143B** | Female,13 years, Caucasian | Thymidine kinase negative (TK-). | Berson JF et al. 1996 (2) |
| **SaOS2** | Female, 11 years, Caucasian | Hypotriploid with the modal number of 56 chromosomes per cell | Banerjee C et al. 1996 (1) |
| **U2OS** | Female, 15 years, Caucasian | Highly altered chromosomes | Heldin CH et al. 1986 (4) |

# Supplemental Figure S1

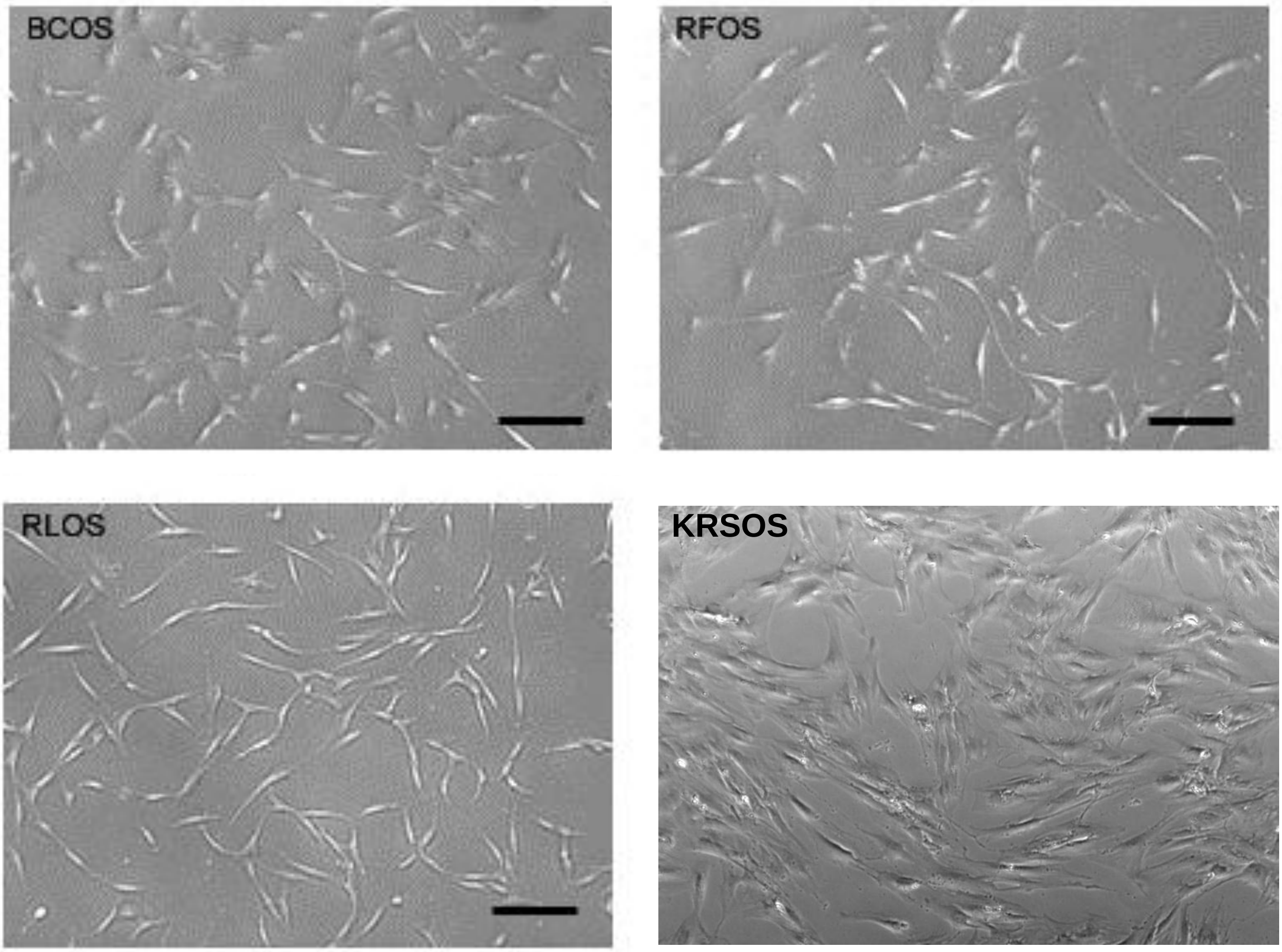

**Supplemental Figure S2**

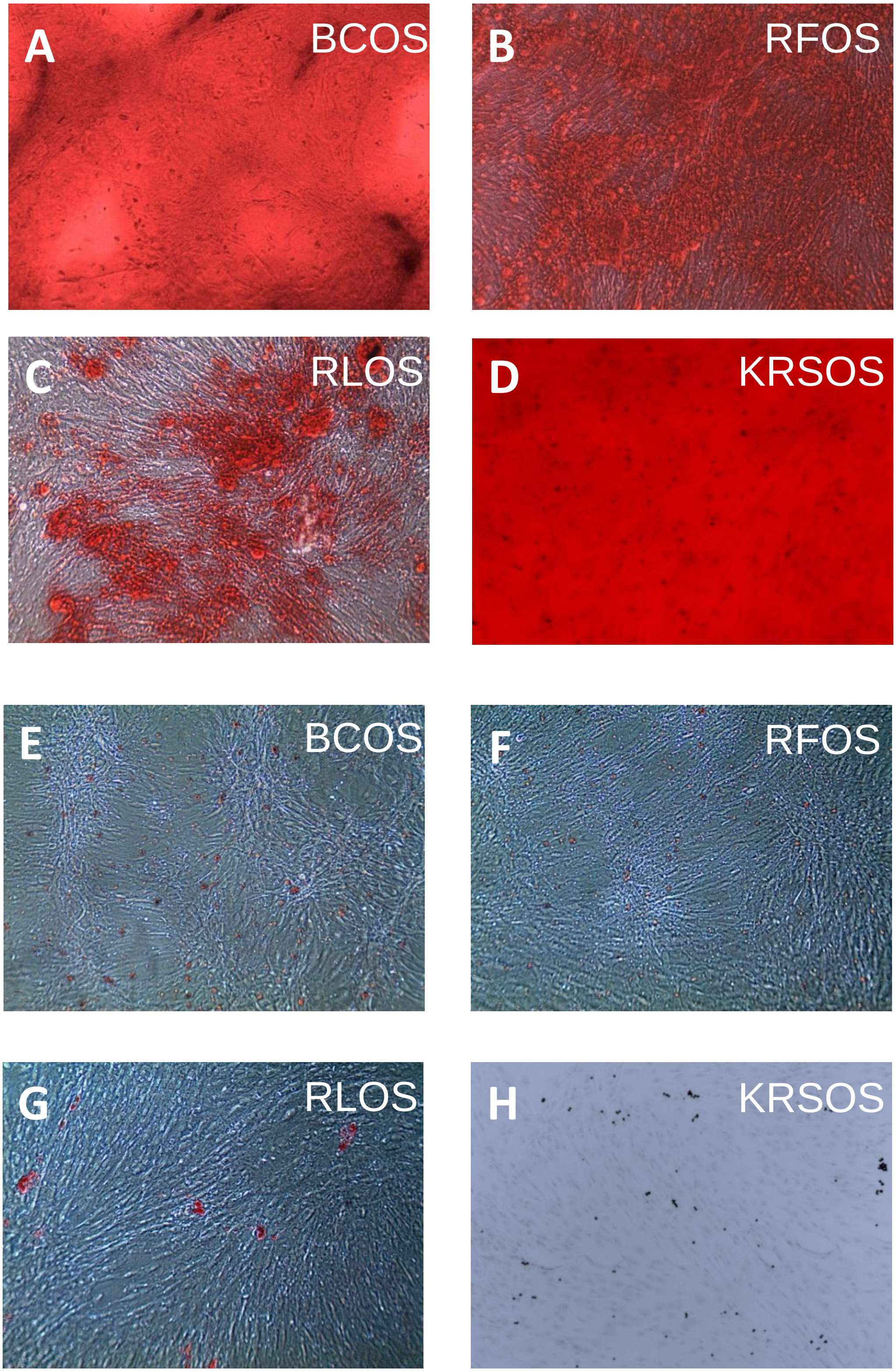

**References list for Table S1:**

1. Banerjee C, Hiebert SW, Stein JL, Lian JB, Stein GS 1996 An AML-1 consensus sequence binds an osteoblast-specific complex and transcriptionally activates the osteocalcin gene. Proc Natl Acad Sci U S A **93**(10)**:**4968-73.
2. Berson JF, Long D, Doranz BJ, Rucker J, Jirik FR, Doms RW 1996 A seven-transmembrane domain receptor involved in fusion and entry of T-cell-tropic human immunodeficiency virus type 1 strains. J Virol **70**(9)**:**6288-95.
3. Billiau A, Edy VG, Heremans H, Van Damme J, Desmyter J, Georgiades JA, De Somer P 1977 Human interferon: mass production in a newly established cell line, MG-63. Antimicrob Agents Chemother **12**(1)**:**11-5.
4. Heldin CH, Johnsson A, Wennergren S, Wernstedt C, Betsholtz C, Westermark B 1986 A human osteosarcoma cell line secretes a growth factor structurally related to a homodimer of PDGF A-chains. Nature **319**(6053)**:**511-4.
5. McAllister RM, Gardner MB, Greene AE, Bradt C, Nichols WW, Landing BH 1971 Cultivation in vitro of cells derived from a human osteosarcoma. Cancer **27**(2)**:**397-402.
6. Oliner JD, Kinzler KW, Meltzer PS, George DL, Vogelstein B 1992 Amplification of a gene encoding a p53-associated protein in human sarcomas. Nature **358**(6381)**:**80-3.
7. Rhim JS, Cho HY, Huebner RJ 1975 Non-producer human cells induced by murine sarcoma virus. Int J Cancer **15**(1)**:**23-9.
8. Roberts WM, Douglass EC, Peiper SC, Houghton PJ, Look AT 1989 Amplification of the gli gene in childhood sarcomas. Cancer Res **49**(19)**:**5407-13.
